# Are Nutrition Standards for Beverages in Schools Associated with Healthier Beverage Intakes among Adolescents in the US?

**DOI:** 10.3390/nu13010075

**Published:** 2020-12-29

**Authors:** Jamie F. Chriqui, Julien Leider, Juliana F. W. Cohen, Marlene Schwartz, Lindsey Turner

**Affiliations:** 1 Division of Health Policy and Administration, School of Public Health, University of Illinois Chicago, Chicago, IL 60612, USA; 2 Institute for Health Research and Policy, University of Illinois Chicago, Chicago, IL 60608, USA; jleide2@uic.edu; 3 Department of Public Health and Nutrition, Merrimack College, 315 Turnpike Street, North Andover, MA 01845, USA; cohenj@merrimack.edu; 4 Department of Nutrition, Harvard T.H. Chan School of Public Health, 677 Huntington Ave, Boston, MA 02115, USA; 5 Rudd Center for Food Policy and Obesity, Department of Human Development and Family Sciences, University of Connecticut, 1 Constitution Plaza, Hartford, CT 06103, USA; marlene.schwartz@uconn.edu; 6 College of Education, Boise State University, 1910 University Drive, Boise, ID 83725, USA; lindseyturner1@boisestate.edu

**Keywords:** beverage, school policy, nutrition, dietary intake, Smart Snacks

## Abstract

Under the U.S. Department of Agriculture’s Smart Snacks in School standards, beverages sold in schools are restricted to water, flavored or unflavored non-fat milk or unflavored low-fat milk (and milk alternatives), and 100% fruit and vegetable juices; and, at the high school level, diet (≤10 kcal), low-calorie (≤60 kcal), and caffeinated beverages may also be sold. Using data from the School Nutrition and Meal Cost Study, this study examined whether secondary school student beverage consumption was associated with school-level à la carte and vending machine beverage availability, controlling for district, school, and student characteristics. On average, most beverages sold in middle schools (84.54%) and high schools (74.11%) were Smart Snacks compliant; while 24.06 percent of middle school students and 14.64 percent of high school students reported consuming non-compliant beverages, including non-compliant milk, fruit drinks, and sports or energy drinks. School beverage availability was not related to consumption among middle school students; however, high school students were less likely to consume non-compliant beverages when enrolled in schools that sold a higher proportion of compliant beverages (Range: OR = 0.97–0.98, 95% CI = 0.95, 1.00). Findings from this study build upon prior research illustrating the role that schools can play in influencing student dietary intake.

## 1. Introduction

In the United States, sugary drinks are the primary source of added sugars in the American diet [1,2,3]. Beverages (including soft drinks, fruit drinks, sports and energy drinks, sweetened coffees and teas, and flavored waters) account for 47% of all added sugars in the diet of the U.S. population aged 2 years and older, and intake of added sugars is most prominent among children, adolescents, and young adults [3]. The 2015–2020 Dietary Guidelines for Americans recommend limiting added sugar intake to less than 10% of calories per day; however, rates of added sugar consumption among children and adolescents aged 4 through 18 range from 14 to 18% of daily energy intake, varying by gender and age group [3]. The Dietary Guidelines recommend that Americans reduce the portion size and frequency of their sugary drink consumption, and instead choose unflavored water or beverages with no or low amounts of sugar [3].

**Figure 1:**
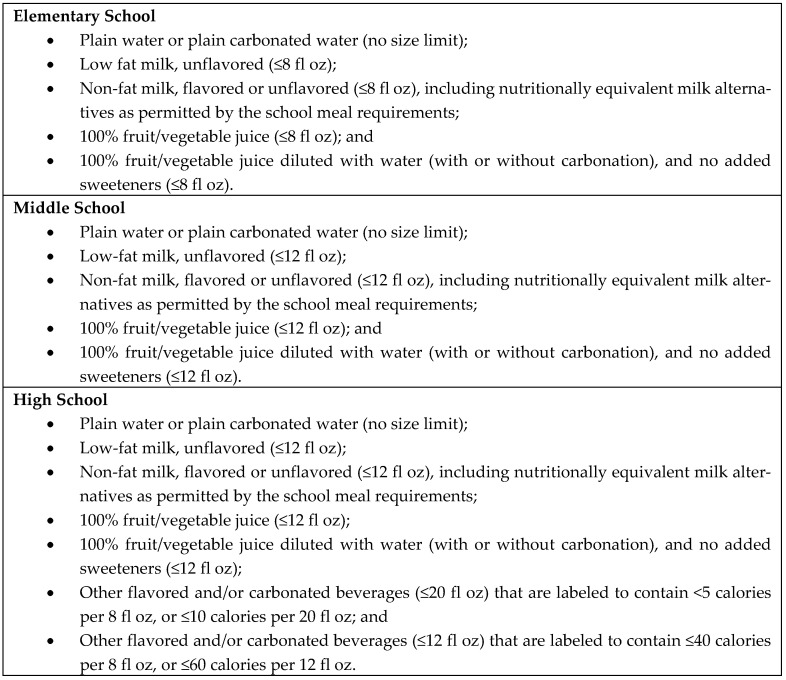
Beverages Allowed as Part of the U.S. Department of Agriculture’s Smart Snacks in School Standards [34,35].

Recognizing that children spend approximately 6 h per day for at least 180 days per year in school in the United States [4] and often consume up to half their daily calories there [4,5], reducing availability and consumption of sugary beverages in schools has been a key population-level intervention and policy strategy for reducing children’s and adolescents’ intake of sugary drinks [6]. Prior to 2014, there was a patchwork of state and local policy strategies governing school food and beverage availability in the United States and restricting the availability of sugary drinks in schools [7,8,9,10]. Research has demonstrated the impact of reducing the availability of sugary drinks in schools through changes in state or local policies on student beverage purchasing and/or consumption [11,12,13,14,15,16,17,18,19,20,21,22,23,24,25,26,27]. Yet, it was not until the Healthy, Hunger-Free Kids Act of 2010 [28] that the U.S. Department of Agriculture (USDA) possessed the statutory authority to regulate foods and beverages sold in schools outside of the school meal programs, which are widely available in most schools throughout the United States (and frequently sold to children internationally through canteens and local vendors) [29,30,31,32,33]. To implement the Act, the USDA issued an interim final rule in June 2013 governing the sale of foods and beverages sold in schools in competition with the National School Lunch Program and the School Breakfast Program (i.e., competitive foods and beverages) [34]. The interim final rule took effect with the beginning of school year 2014–2015; a final rule was issued in July 2016 and renamed the guidelines as the Smart Snacks in School standards [35]. Figure 1 outlines the beverages that are allowed to be sold in schools under the Smart Snacks rule (they are the same standards that applied under the interim final rule).

The purpose of this study was twofold. First, we sought to investigate the availability of beverages that were and were not Smart Snacks compliant in U.S. middle and high schools during the first year of implementation of the standards. Second, we examined whether student consumption of non-compliant beverages was lower when school-level compliance was higher.

## 2. Materials and Methods

This cross-sectional study examined the association of school beverage availability and student beverage consumption, controlling for district policy and school and student characteristics, using data from the School Nutrition and Meal Cost Study (SNMCS) and the National Wellness Policy Study (NWPS). SNMCS data were collected during the 2014–2015 school year and the student-level data are nationally representative of students enrolled in public, non-charter schools participating in the National School Lunch Program. The methodology report for the SNMCS describes in detail the study design, as well as sampling, recruitment, data collection, and data processing procedures [36]. Additional details about collection and analysis of the data used in this paper are available in Volumes 1 and 4 of the SNMCS final report [37,38]. NWPS data on district policies in effect at the beginning of the 2014–2015 school year were collected for the districts in which students were enrolled and coded using established protocols [9]. NWPS data were linked to SNMCS data by Mathematica Policy Research, and de-identified data were returned to the University of Illinois Chicago for analyses. This study was deemed to “not involve human subjects” by the University of Illinois Chicago Institutional Review Board (protocol #2020-0448).

### 2.1. Measures

#### 2.1.1. Student Beverage Consumption

Data on student beverage consumption were obtained from 24 h dietary recalls conducted as part of the SNMCS Child/Youth Interview. Data were collected and processed using USDA’s Dietary Intake Data System, which includes the Automated Multiple-Pass Method (AMPM) computer-assisted personal interview to collect dietary recall data and the Post-Interview Processing System and Survey Net to process the data. The AMPM was modified to incorporate specific school locations, such as the school store and cafeteria line. Middle and high school students completed the dietary recall independently, except for middle school students in schools spanning elementary and middle grades, who completed the recall with parental assistance.

Item-level data from day 1 recalls were used. Only items that were consumed at school and either (1) consumed at breakfast or lunch or (2) obtained at school were counted for purposes of constructing analytical measures. Beverages were classified into the following types, based on the major and minor food groups used for the SNMCS report [38]: water, 100% juice, milk, diet carbonated soda, regular carbonated soda, fruit drinks, nutritional beverages, sports and energy drinks, and tea and coffee. This classification was then used in combination with nutritional data on the calorie and caffeine content of the beverages to categorize each beverage as Smart Snacks compliant or not by grade level. For middle school, water without calories, 100% juice, unflavored skim or 1% milk, and flavored skim milk were counted as compliant as long as they contained no caffeine; all other beverages were counted as non-compliant. For high school, 100% juice, unflavored skim or 1% milk, and flavored skim milk were always counted as compliant, while the following were counted as compliant as long as they fell under specific calorie limits: water, diet carbonated soda, regular carbonated soda, fruit drinks, sports or energy drinks, and tea or coffee. Two different calorie limits of 10 and 60 kcal were used to construct the high school measure, based on Smart Snacks maximum standards of ≤10 kcal per 20 fluid ounces for no calorie beverages and ≤60 kcal per 12 fluid ounces for low-calorie beverages. Data were only available on portion sizes by weight (in grams) and not volume (e.g., fluid ounces), so it was not possible to incorporate beverage size into the compliance measure. All nutritional beverages (e.g., protein drinks) were counted as non-compliant for both middle and high school based on their calorie content, and a small number of “other milk beverages” were excluded as we lacked data on their milk fat percentage; only 4 and 8 item-level observations were affected, respectively, out of 1703 total for middle and high school. Student-level measures were constructed based on these item-level measures to indicate whether each student consumed non-compliant beverages (yes/no).

#### 2.1.2. School Beverage Availability

Data on school beverage availability were obtained from the SNMCS À la Carte and Vending Machine Checklists and used to determine the proportion of available beverages that were Smart Snacks compliant [39]. The À la Carte Checklist included separate line items for 16 specific beverages at breakfast and lunch (other write-in beverages were not considered for these analyses): whole, reduced-fat, low-fat, and fat-free white milk; reduced-fat, low-fat, and fat-free flavored milk; 100% juice; bottled water; diet carbonated soft drinks; regular carbonated soft drinks; juice drinks and other sweetened drinks; sports drinks; energy drinks; hot or cold chocolate drinks; and hot or cold coffee or tea. Responses from the À la Carte Checklist were coded as missing for non-milk items where the respondent indicated that à la carte items other than milk were served at breakfast or lunch, but no specific non-milk items were selected. Responses were also coded as missing (i.e., not applicable) for all breakfast items where the school did not serve breakfast. The Vending Machine Checklist included separate checklists for each vending machine available to students, including before or after school, and asked respondents to provide information for each machine on the availability of a number of specific items. Binary (yes/no) indicators were constructed for whether each of 13 specific beverages was available in any machine in the given school (as with à la carte, other write-in beverages were not considered for these analyses): whole or reduced-fat, low-fat, and fat-free white milk; whole or reduced-fat, low-fat, and fat-free flavored milk; diet carbonated soft drinks; regular carbonated soft drinks; 100% juice; juice drinks and other sweetened drinks; energy and sports drinks; bottled water; and hot or cold chocolate drinks. In computing the proportion of available beverages across à la carte and vending that were Smart Snacks-compliant, low-fat and fat-free white milk, fat-free flavored milk, 100% juice, and bottled water were counted as compliant in both middle and high school, while diet carbonated soft drinks were counted as compliant in high school only; all other beverages were counted as non-compliant. Because the checklists did not include specific nutritional characteristics, the classification of items as compliant or non-compliant was made based on item descriptions alone. Sensitivity analyses treating hot or cold coffee or tea as compliant for high school were conducted and are described below. Items for which availability was missing were excluded from the denominator for purposes of computing the proportion compliant. In cases where no beverages were available and availability was non-missing for at least some, the proportion compliant was recoded as 100%.

#### 2.1.3. Policy

All school districts participating in the National School Lunch Program were required as of the beginning of school year 2006–2007 to adopt and implement a wellness policy that includes guidelines for all food and beverages sold in competition with the school meal programs [28,40]; therefore, we also controlled for district-level policies governing school beverage availability in à la carte and vending machine settings for school year 2014–2015 in each of the districts where the SNMCS schools were located. District policies were coded using the National Wellness Policy Study measure [41] for nutrition standards for beverages available from à la carte and vending in middle schools (item NS25) and high schools (item NS26). A binary (yes/no) indicator of whether the district policy met Smart Snacks in both à la carte and vending machines was computed separately for middle and high school, based on whether NS25 (middle school) was coded at level 3 or above (i.e., met Smart Snacks standards or complete ban on competitive beverages or a ban on sales in competitive venues) or NS26 (high school) was coded at level 2 or above (i.e., met Smart Snacks standards or complete ban on competitive beverages or a ban on sales in competitive venues), respectively, in both venues.

#### 2.1.4. Control Variables

Data on student-level and school-level characteristics were obtained from SNMCS and the National Center for Education Statistics [42]. Student-level characteristics included grade level (continuous variable), gender (male or female), race/ethnicity (non-Hispanic white, non-Hispanic Black, Hispanic, or other, including multi-racial), and household income as a percentage of the poverty level (categorized as ≤130%, >130–185%, and >185%, based on thresholds for free/reduced-price lunch eligibility). School-level characteristics included the school grade level (middle or high school, used for stratification), student racial/ethnic distribution (categorized as ≥50% non-Hispanic white, ≥50% non-Hispanic Black, ≥50% Hispanic, and other), the percentage of students eligible for free or reduced-price lunch (categorized by tertiles as 0.00–37.42%, >37.42–63.37%, and >63.37–100.00%), size (<500, 500–999, or ≥1000 students), and urbanicity (urban, suburban, or rural). Region was determined for each school based on state using Census definitions (West, Midwest, South, or Northeast) [43].

### 2.2. Sample

Dietary recalls were completed by 714 middle school and 703 high school students. Data on the proportion of à la carte and vending beverages that were Smart Snacks compliant were missing for 8 middle school students, data on student race/ethnicity or household income were missing for 187 middle school and 189 high school students, and data on other school or student characteristics were missing for 5 middle school and 4 high school students. The final analytical sample included 514 middle school students in 90 middle schools and 510 high school students in 92 high schools located in 92 SFAs and 30 states and DC. The final analytical samples for middle and high school both included 1–9 students per school, consistent with the target of 8 students per school for the SNMCS sample design, which was chosen to generate a large enough sample of students for nationally representative estimates [36]. The final analytical sample was similar to the original sample of students with the following exceptions: (1) for district policy at the high school level, 32.79% of students in the analytical sample were in a district with a policy compared to 50.90% of students who had to be excluded; (2) for school urbanicity at the middle school level, 22.16% of the analytical sample were in rural areas compared to 36.20% for excluded students; (3) for student race at the high school level, 56.23% of the analytical sample was non-Hispanic white vs. 75.58% for excluded students; and (4) for student household income at >185% of the poverty threshold, 61.35% of middle school students’ households in the analytical sample were at this threshold compared to 43.56% for excluded students and 67.86% of high school students’ households in the analytical sample were at this threshold compared to 33.16% for excluded students.

### 2.3. Statistical Analysis

Multi-variable logistic regression models were computed examining the association between student consumption of non-Smart Snacks-compliant beverages (at both the 10 and the 60 kcal thresholds which are akin to diet and low-calorie beverages, respectively) and the proportion of à la carte and vending machine beverages that were Smart Snacks compliant, controlling for district policy meeting Smart Snacks for beverages in à la carte and vending machines, as well as school- and student-level characteristics. Models were computed separately for middle school and high school students, based on school grade level. Adjusted prevalence estimates were computed from these models by the proportion of Smart Snacks-compliant à la carte and vending machine beverages, at values ranging from 0% to 100% (in intervals of 10%) Smart Snacks-compliant beverages. Analyses were conducted in Stata/SE (version 15.1, StataCorp LP, College Station, TX, USA; 2016) accounting for the survey design and weights.

## 3. Results

### 3.1. Sample Characteristics

Characteristics of the analytic sample by school level are presented in Table 1. Student-reported consumption of Smart Snacks non-compliant beverages at both the 10 and 60 kcal thresholds was higher at the middle school levels than at the high school levels (due primarily to fruit drinks and flavored milks); while school-level compliance with the Smart Snacks standards was higher at the middle than at the high school levels. On average, 46.6% of middle school students and 32.8% of high school students were enrolled in a school located in a district with a policy that met Smart Snacks beverage standards. The majority of students were enrolled in a school with a majority of non-Hispanic white students (60.1% at the middle school level and 70.8% at the high school level); however, nearly 40% of middle school students and nearly 30% of high school students were enrolled in schools that were not majority white. The majority of students were enrolled in schools with low to medium free- and reduced-price lunch eligibility rates and most schools enrolled 500 students or more. The schools were located in urban, suburban and rural areas and all four census regions. The sample was equally divided by male and female gender and 54% of middle school students and 56% of high school students were non-Hispanic white, while the remaining 44–46% of students were non-white. Over 60% of the students were from a family with a household income of >185% of the federal poverty level.

### 3.2. Availability of Smart Snacks-Compliant and Non-Compliant Beverages in Schools

Overall, middle school and high school students attended a school with a high rate of compliance with the Smart Snacks beverage standards, on average. At the middle school level, 84.54% of beverages sold were compliant (Table 1) and 15.46% were non-compliant. At the high school level, 74.11% of the beverages sold were compliant (Table 1), while 25.89% were non-compliant.

Table 2 presents data on the weighted percentage of students located in middle and high schools with access to specific Smart Snacks-compliant and non-compliant beverages in à la carte and vending machine locations. Student exposure to compliant beverages was fairly comparable across school levels for à la carte settings with the exception of fat-free/skim flavored milk at breakfast and lunch, 100% fruit/vegetable juice at breakfast, bottled water at breakfast, and low-fat white milk at lunch (with student access higher at the high school levels in each of these instances). Student access to Smart Snacks-compliant beverages in vending machines was also greater at the high school level than at the middle school level, likely due to less availability of vending machines in middle schools (only 45.7% of middle school students had access to vending machines in school as compared to 88.2% of high school students). Non-compliant beverages available in middle and high schools primarily included juice drinks and other sweetened drinks, regular soda, sports drinks, coffees or teas, whole or reduced-fat white or flavored milk, and low-fat flavored milk.

### 3.3. Student Consumption of Smarts Snacks-Compliant and Non-Compliant Beverages

The Smart Snacks-compliant beverages most commonly consumed by students at school were water, milks, and 100% juices (Table 3). The most commonly consumed non-compliant beverages at the middle school level were non-compliant milks and fruit drinks. At the high school level, fruit drinks, sports or energy drinks, and teas and coffees were the most commonly consumed non-compliant beverages.

### 3.4. Association between School Availability of Compliant Beverages and Student Consumption of Non-Compliant Beverages

**Figure 2:**
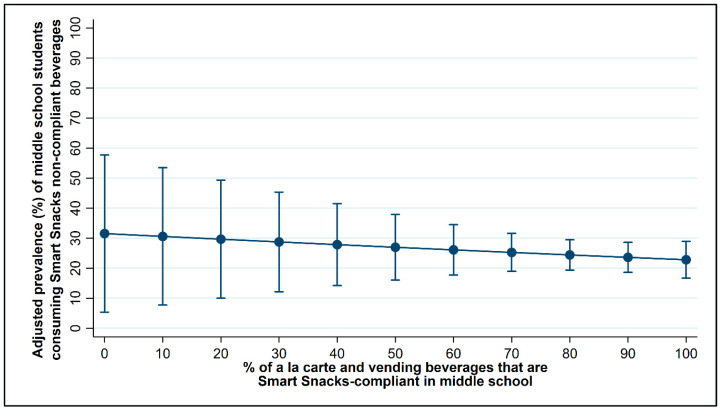
Adjusted prevalence of middle school student consumption of non-compliant beverages in school by the percentage of compliant beverages sold in school.

**Figure 3:**
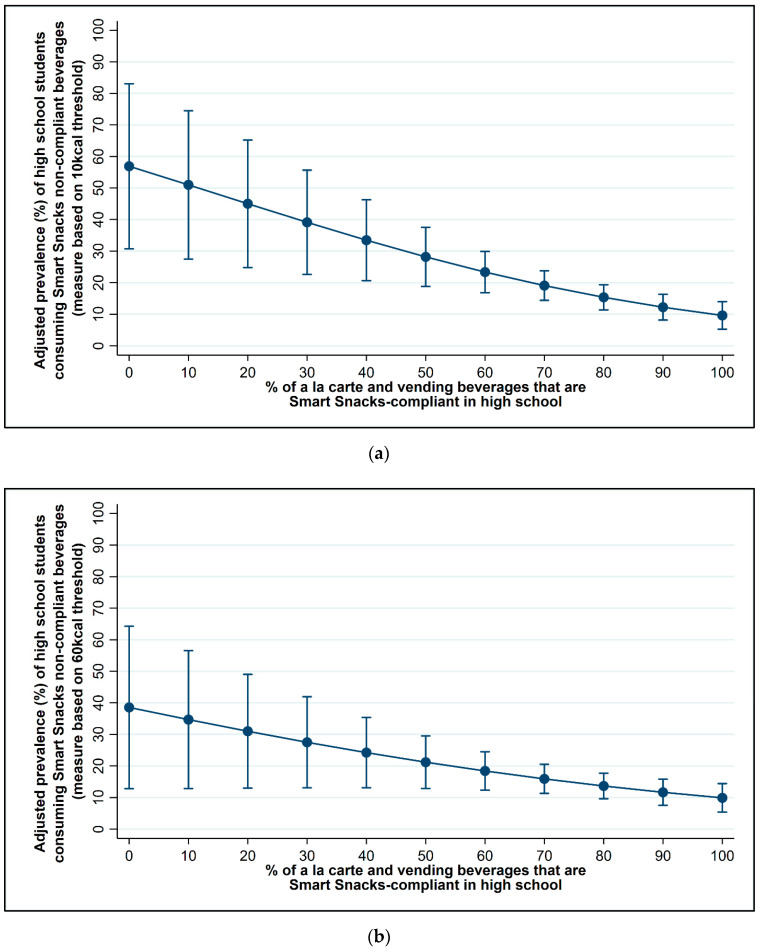
Adjusted prevalence of high school student consumption of non-compliant beverages in school by the percentage of compliant beverages sold in school. The two panels model the adjusted prevalence of student consumption of Smart Snacks non-compliant beverages, after controlling for district, school, and student characteristics at the (**a**) 10 and (**b**) 60 kcal thresholds.

Figure 2 and Figure 3 and Table A1 and Table A2 present the results of the multi-variable logistic regression models showing the association between the percent of beverages sold in schools that were Smart Snacks compliant and student consumption of non-compliant beverages while in school at the middle and high school levels, respectively, controlling for district policy, school, and student characteristics. (Descriptive statistics on characteristics of students who did and did not consume non-compliant beverages are shown in Table A3 for reference.)

There was not an association between beverage availability and student consumption at the middle school level (Figure 2; Table A1). Yet, in the adjusted models (Table A1), middle school students had lower odds of consuming non-compliant beverages if they were enrolled in a school that had ≥50% Hispanic students (OR: 0.25, 95% CI: 0.08, 0.75) compared to a school that had ≥50% non-Hispanic white students and higher odds of consumption if they were enrolled in a school with a high compared to low FRPL eligibility rate (OR: 4.59, 95% CI: 1.85, 11.38).

At the high school level, however, there was a significant, inverse relationship between the percentage of Smart Snacks-compliant beverages sold in schools and student consumption of non-compliant beverages while in school (see Figure 3 and Table A1 and Table A2). Specifically, the odds of students consuming Smart Snacks non-compliant beverages in school at the 10 and 60 kcal thresholds was significantly lower in schools with a higher percentage of Smart Snacks-compliant beverages (10 kcal threshold: OR: 0.97, 95% CI: 0.95, 0.99, *p* < 0.01; 60 kcal threshold: OR: 0.98, 95% CI: 0.96, 1.00, *p* < 0.05). Figure 3 illustrates the adjusted prevalence of high school student consumption of non-compliant beverages at the 10 kcal (panel a) and 60 kcal (panel b) thresholds as the percentage of compliant beverages available in schools increases from 0 to 100%. At the 10 kcal threshold (Figure 3, panel a), if none of the beverages in high school were Smart Snacks compliant, the adjusted prevalence of student consumption of non-compliant beverages at school is estimated to be nearly 60%; whereas, if 100% of the beverages sold in high school were compliant, the estimated adjusted prevalence of student consumption of non-compliant beverages at school is only 10% (brought from home or from a friend perhaps). Similarly, at the 60 kcal threshold (Figure 3, panel b), if none of the beverages sold in high school were Smart Snacks compliant, it is estimated that slightly less than 40% of students would consume non-compliant beverages at school; whereas, if 60% of the beverages sold in high school were compliant, slightly less than 20% of students would be estimated to consume non-compliant beverages at school.

The adjusted models (Table A1 and Table A2) also revealed that high school students had lower odds of consuming non-compliant beverages at the 10 kcal threshold (i.e., diet beverages) if they were in a school with a diverse racial/ethnic mix (compared to majority non-Hispanic white) of students (OR: 0.33, 95% CI: 0.15, 0.73), from a medium-sized school (as compared to a large school) (OR: 0.27, 95% CI: 0.11, 0.65), or from a household with an income that was ≤130% of the federal poverty level (OR: 0.34, 95% CI: 0.14, 0.83) or at >130–185% of the federal poverty level (OR: 0.11, 95% CI: 0.03, 0.44) as compared to a household with an income >185% of the federal poverty level. Similar results were found for the 60 kcal (lower calorie) beverage threshold (albeit with slightly different coefficients and confidence intervals; see Table A2), except that students enrolled in schools with high rates of FRPL eligibility had higher odds than high school students enrolled in schools with low rates of FRPL eligibility of consuming non-compliant beverages at the 60 kcal threshold (OR: 3.31, 95% CI: 1.20, 9.13).

In additional sensitivity analyses (not shown in tables), we added body mass index (BMI) status as a covariate, based on BMI-for-age percentiles computed based on age, gender, and measured height and weight with the following categories: underweight or healthy weight (<85th percentile); overweight (≥85th–<95th percentile); and obese (≥95th percentile). Results were similar to those in our primary models; for BMI status itself, we found that obese middle school students were less likely to consume non-compliant beverages than underweight or healthy weight middle school students (OR: 0.46, 95% CI: 0.24, 0.90), although no significant differences were found for overweight middle students compared to underweight or healthy weight middle school students or for either category relative to underweight or healthy weight high school students. Due to missing data, these analyses included 489 middle school and 478 high school students.

## 4. Discussion

To our knowledge, this was the first study to examine the availability and consumption of Smart Snacks-compliant and non-compliant beverages and student consumption of non-compliant beverages while in school across the United States. Although the observed rates of compliance with the Smart Snacks beverage standards were high, there was not universal compliance, which is consistent with studies conducted in Massachusetts that examined a comparable state beverage policy that was in place prior to the national standards [12,13]. Furthermore, previous qualitative research conducted among food service directors found that compliance with competitive food policies varied for several reasons. Food service directors indicated that ensuring that competitive foods aligned with the multiple aspects of the updated standards (including calories, caffeine levels, and portion sizes that varied by grade level) could be challenging, especially without sufficient training, resources, and/or support from the school district. Food service directors reported that it was easier to comply with the standards when reference lists for compliant foods were used (e.g., John C. Stalker A-List), when the district had strong wellness policies, and when they were able to work with their district’s wellness committee to ensure compliant foods were sold [44].

In the present study, the average percentage of Smart Snacks-compliant beverages sold in à la carte and vending machine locations in middle and high schools was high: 84.5% at the middle school level and 74.1% at the high school level. This is encouraging because the data collected for this study are from the first year of implementation of the Smart Snacks standards (i.e., school year 2014–2015). This is consistent with prior research which similarly found high rates of compliance with competitive food policy changes in the first year of implementation [13].

It was encouraging that less than 25% of middle school students, and approximately only 1 in 6 high school students, reported consuming non-compliant beverages while in school during the time of the study. With a few exceptions, student in-school consumption of non-compliant beverages did not vary greatly by school, student, or household characteristics. Because of the cross-sectional nature of this study; it will be important to continue to monitor student beverage consumption (in and out of school) over time to understand the factors that are the greatest contributors to their consumption of beverages higher in sugars, calories, and/or fat (milk fat).

Student in-school consumption of non-compliant beverages was lower for high school students attending schools where a higher proportion of compliant beverages were sold. This is consistent with prior literature that found an association between strong beverage standards and reduced student consumption of unhealthy beverages [19,26]. This reduction in in-school unhealthy beverage consumption may have important health implications for students, as previous research has documented that students do not compensate for only having access to healthier beverages in school by consuming more unhealthy drinks after school [27]. At the middle school level, however, we did not see any association between compliant beverage availability and student consumption of non-compliant beverages. This is inconsistent with prior research findings [11,12,45] and may be in part due to the lack of precision of the nutrient profiles of the beverages sold in schools and reported in the à la carte and vending machine checklists in the present study. On the other hand, the lack of an association between compliant beverage availability and consumption of non-compliant beverages may have also been due to the substantially fewer competitive food options typically available in middle schools compared with high schools. It is possible that having a greater variety of healthy beverage choices increases the likelihood of their selection and is an area for future study [13,14].

The current findings highlight a few notable areas of concern in terms of the availability and consumption of specific non-compliant beverages in school. First, flavored milks are a contentious topic within the nutrition and public health worlds. While the Smart Snacks standards do not allow flavored low-fat, reduced fat, or whole milk, the standards do allow flavored non-fat (skim) milk. From a dietary intake perspective, this approach helps reduce consumption of solid fats, but it does not reduce the consumption of added sugars: eight ounces of flavored milk contains on average half of a child’s recommended maximum intake of added sugar per day, and flavored milks are a top contributor of sugar intake in schools [46,47]. Second, although carbonated soda and soft drink consumption among youth has declined, the consumption of sports drinks, energy drinks and fruit drinks remains high [48,49]. Indeed, in the present study, those beverages (along with flavored skim milk containing caffeine at the middle school level only) accounted for the largest percentage of non-compliant beverages sold in schools and consumed by students while in school. This highlights the importance of providing and promoting lower sugar beverage options in schools, such as plain or sparkling water. When healthier alternatives are available, research has found that similar policy changes are associated with significant reductions in overall sugar consumption [27].

### Study Limitations

This study should be viewed in the context of the following limitations. First, this was a cross-sectional study conducted at one point in time (school year 2014–2015). As a result, significant findings represent associations but not causation and future studies should examine secular trends in changes in beverage availability and student consumption over a longer time horizon. Second, we lacked nutritional information on competitive beverages available à la carte and in vending machines, and had to rely instead on classifying these beverages as compliant or non-compliant based on the broad descriptions used in the SNMCS À la Carte and Vending Machine Checklists [39]. This was particularly ambiguous for hot or cold coffee or tea offered à la carte, for which we could not know whether the coffee/tea was served pre-sweetened. For our main analyses, we treated this as non-compliant, and our key findings were unchanged in sensitivity analyses where this was treated as compliant at the high school level. Third, we only had portion size information in grams for beverages consumed. Thus, we were unable to assess portion sizes in fluid ounces, which is necessary to determine compliance with the calorie limits of the Smart Snacks standards. To address this, we conservatively used the higher thresholds for diet (10 kcal/20 fluid ounces) and low-calorie (60 kcal/12 fluid ounces) beverages to determine compliance and conducted sensitivity analyses at both thresholds. Fourth, as is true of all research based on dietary recalls, the student beverage consumption measures used in this study are subject to error from self-reporting as well as error in coding beverage types and nutritional content. Fifth, the analytical sample was relatively higher-income due to exclusions for missing data; associations may be different for lower-income students and this is an area for future study. Finally, this study specifically analyzed student beverage consumption at school and did not analyze consumption outside of school, and future studies should also explore whether secondary school students’ overall consumption (including in-school and out-of-school consumption) of less healthy beverages (akin to the Smart Snacks non-compliant beverages) declines over time.

## 5. Conclusions

In summary, this is the first large-scale study to examine the association between the availability of beverages sold through à la carte and vending machines in school and student beverage consumption. With more compliant beverages and fewer non-compliant beverage options available in schools, high school students were significantly less likely to consume less healthy drinks. This study adds to the growing evidence that strong school nutrition policies have the ability to improve the nutritional intake and diets of students.

## Figures and Tables

**Table 1:** Survey-weighted characteristics of the analytic sample by school level.

Variable	Middle School % or Mean (95% CI)	High School % or Mean (95% CI)
Student-level outcome variables		
Student consumed SS non-compliant beverage (10 kcal threshold) *	24.06 (19.61, 29.15)	17.23 (13.61, 21.57)
Students consumed SS non-compliant beverage (60 kcal threshold) *	24.06 (19.61, 29.15)	14.64 (11.11, 19.06)
% of à la carte and vending machine beverages sold in school that are SS compliant (school predictor) (mean)	84.54 (78.05, 91.03)	74.11 (70.22, 78.00)
District policy meets SS for à la carte and vending machine beverages	46.64 (32.48, 61.37)	32.79 (21.41, 46.64)
School-level race/ethnicity of students		
≥50% NH white	60.11 (45.16, 73.39)	70.75 (57.08, 81.47)
≥50% NH Black	4.03 (0.91, 16.11)	4.84 (1.96, 11.42)
≥50% Hispanic	12.66 (6.35, 23.67)	6.48 (2.89, 13.89)
Mixed	23.19 (12.77, 38.38)	17.93 (9.20, 32.04)
School-level FRPL eligibility rates		
Low (0.00–37.42)	39.03 (25.97, 53.87)	58.37 (44.79, 70.78)
Medium (>37.42–63.37)	38.94 (25.70, 54.04)	25.29 (16.09, 37.41)
High (>63.37–100.00)	22.03 (12.93, 34.97)	16.34 (9.31, 27.11)
School size		
Small (fewer than 500 students)	18.19 (9.89, 31.06)	7.42 (3.33, 15.72)
Medium (500 to 999 students)	55.46 (41.55, 68.56)	20.72 (12.21, 32.92)
Large (1000 or more students)	26.35 (16.52, 39.27)	71.86 (59.76, 81.45)
School urbanicity		
Urban	30.80 (18.95, 45.87)	21.23 (12.02, 34.71)
Suburban	47.04 (32.92, 61.64)	54.47 (40.59, 67.69)
Rural	22.16 (13.05, 35.07)	24.30 (14.34, 38.11)
Student-level gender		
Male	50.00 (45.37, 54.63)	50.57 (44.95, 56.17)
Female	50.00 (45.37, 54.63)	49.43 (43.83, 55.05)
Student-level race/ethnicity		
White, non-Hispanic	54.28 (45.10, 63.18)	56.23 (48.98, 63.21)
Black, non-Hispanic	11.66 (6.54, 19.93)	13.58 (9.25, 19.49)
Hispanic	22.64 (15.74, 31.43)	19.86 (14.81, 26.10)
Other (including multi-racial)	11.42 (8.22, 15.65)	10.34 (7.32, 14.41)
Household income as a percentage of poverty level		
≤130%	29.64 (23.24, 36.95)	22.72 (17.46, 29.01)
>130–185%	9.01 (6.55, 12.29)	9.42 (6.64, 13.20)
>185%	61.35 (53.79, 68.39)	67.86 (60.24, 74.63)
Census region		
West	14.40 (7.54, 25.78)	11.94 (5.89, 22.70)
Midwest	27.97 (16.67, 43.00)	31.26 (19.43, 46.17)
South	44.96 (30.81, 59.99)	43.21 (29.77, 57.74)
Northeast	12.66 (6.09, 24.47)	13.58 (6.32, 26.81)

Notes: Weighted percentages or means were computed taking into account the SNMCS 2014–2015 sampling design. *n* = 514 middle school students and *n* = 510 high school students. * Measure only counts consumption where item was consumed at school and either (1) consumed at breakfast or lunch or (2) obtained at school. SS: Smart Snacks. NH: non-Hispanic. FRPL: free/reduced-price lunch.

**Table 2:** Weighted Percentage of Students with Access to Given Smart Snacks-Compliant and Non-Compliant Beverages Sold by Location of Sale and School Level.

Beverage	MS	HS	Beverage	MS	HS
**SS-Compliant À la Carte Beverages Sold**			**SS Non-Compliant À la Carte Beverages Sold**		
Low-fat (≤1%) white milk (br)	67.4	69.6	Whole white milk (br)	0.4	3.2
Fat-free/skim milk (br)	42.4	45.0	Reduced fat (2%) white milk (br)	0.4	7.9
Fat-free/skim flavored milk (br)	57.1	67.3	Reduced fat (2%) flavored milk (br)	1.0	3.9
100% fruit/vegetable juice (br)	53.8	63.5	Low-fat (≤1%) flavored milk (br)	11.2	9.7
Plain, flavored, or sparkling bottled water (br)	47.2	61.2	Juice drinks/other sweetened drinks (br)	4.3	11.0
Diet carbonated soft drinks/soda/diet pop (br)	NC	8.2	Sports drinks (Gatorade/Powerade) (br)	0.0	20.6
Low-fat (≤1%) white milk (lunch)	73.6	83.7	Hot or cold chocolate drinks (not chocolate milk) (br)	0.0	4.3
Fat-free/skim milk (lunch)	46.1	48.0	Hot or cold coffee or tea (br)	0.0	17.8
Fat-free/skim flavored milk (lunch)	61.1	77.0	Whole white milk (lunch)	1.1	3.1
100% fruit/vegetable juice (lunch)	74.2	71.5	Reduced fat (2%) milk (lunch)	1.4	3.4
Plain, flavored, or sparkling bottled water (lunch)	73.3	78.3	Reduced fat (2%) flavored milk (lunch)	1.5	3.7
Diet carbonated soft drinks/diet soda/diet pop (lunch)	NC	13.0	Low-fat (≤1%) flavored milk (lunch)	23.5	15.0
			Juice drinks/other sweetened drinks (lunch)	8.4	11.6
**SS-Compliant Vending Machine Beverages Sold**			Sports drinks (Gatorade/Powerade) (lunch)	5.5	47.6
Diet carbonated soft drinks/diet soda/diet pop	NC	68.7	Hot or cold chocolate drinks (not chocolate milk) (lunch)	3.4	5.0
100% fruit/vegetable juice	23.6	62.1	Hot or cold coffee or tea (lunch)	0.7	29.4
Plain, flavored, or sparkling bottled water	39.8	81.5			
Fat-free/skim flavored milk	0.0	19.3	**SS Non-Compliant Vending Machine Beverages Sold**		
Low-fat (1%) white milk	2.0	9.9	Diet carbonated soft drinks/diet soda/diet pop	14.7	C
			Regular carbonated soft drinks/regular soda/regular pop	14.2	42.3
			Juice drinks/other sweetened drinks	15.9	58.6
			Energy and sports drinks	15.5	75.5
			Hot or cold chocolate drinks (not chocolate milk)	0.0	8.5
			Whole or reduced fat (2%) flavored milk	0.0	13.6
			Low-fat (1%) flavored milk	2.2	7.5
			Whole or reduced fat (2%) white milk	2.0	20.7

Notes: Weighted percentages were computed taking into account the SNMCS 2014-15 sampling design. *n* = 307–474 middle school students and 144–485 high school students due to item-specific missing data. Hot or cold coffee or tea was counted as a non-compliant beverage as it was assumed to be sweetened. MS: middle school. HS: high school. SS: Smart Snacks. BR: breakfast. NC: Non-compliant for middle school grade level. C: Compliant for high school grade level.

**Table 3:** Weighted percentage of students consuming Smart Snacks-compliant and non-compliant beverages at school by school level and kcal threshold *.

Variable	Middle School	High School Threshold	
		10 kcal	60 kcal
**Compliant Beverages**			
Water	39.3	47.6	47.6
Milk	20.1	24.0	24.0
100% juice	17.7	16.6	16.6
Diet carbonated soda	0.0	1.9	2.7
Regular carbonated soda	0.0	0.0	0.1
Fruit drinks	0.0	0.1	1.7
Sports or energy drinks	0.0	0.0	0.3
Tea or coffee	0.0	0.4	1.0
**Non-Compliant Beverages**			
Water	0.4	0.9	0.9
Milk (any non-compliant)	10.0	0.3	0.3
Flavored milk (non-skim)	0.5	0.0	0.0
Flavored milk (skim) with caffeine	8.7	0.0	0.0
Unflavored reduced fat (2%) milk	0.1	0.2	0.2
Unflavored whole milk	0.7	0.1	0.1
Diet carbonated soda (any for middle school or exceeding kcal threshold for high school)	0.5	0.8	0.0
Regular carbonated soda	1.6	2.3	2.2
Fruit drink	7.8	5.5	3.9
Nutritional beverage	0.3	0.5	0.5
Sport or energy drinks	2.6	4.1	3.8
Tea or coffee	1.6	4.5	4.0

Notes: Weighted percentages were computed taking into account the SNMCS 2014–2015 sampling design. *n* = 514 middle school students and *n* = 510 high school students. Measures only count consumption where item was consumed at school and either (1) consumed at breakfast or lunch or (2) obtained at school. For middle school, water without calories, 100% juice, unflavored skim or 1% milk, and flavored skim milk were counted as compliant as long as they contained no caffeine. For high school, 100% juice, unflavored skim or 1% milk, flavored skim milk, and any of the following with at most 10 or 60 kcal were counted as compliant: water, diet carbonated soda, regular carbonated soda, fruit drinks, sports or energy drinks, and tea or coffee. * The kcal thresholds only applied at the high school level.

## Data Availability

Requests for access to the public use SNMCS data should be submitted via electronic mail to: FNSStudies@usda.gov.

## Appendix A

**Table A1:** Logistic regression results predicting student consumption of Smart Snacks non-compliant beverages at the 10 kcal threshold by school level.

Variable	Middle School OR (95% CI)	High School OR (95% CI)
% of à la carte and vending machine beverages sold in school that are SS compliant	1.00 (0.98,1.01)	0.97 ** (0.95, 0.99)
District policy meets SS for à la carte and vending machine beverages	1.02 (0.58, 1.77)	1.22 (0.71, 2.10)
School-level race/ethnicity of students		
≥50% NH White	Referent	Referent
≥50% NH Black	0.15+ (0.02, 1.13)	0.77 (0.18, 3.33)
≥50% Hispanic	0.25 * (0.08, 0.75)	0.38 (0.10, 1.54)
Mixed	0.55 (0.26, 1.15)	0.33 ** (0.15, 0.73)
School-level FRPL eligibility rates		
Low (0.00–37.42)	Referent	Referent
Medium (>37.42–63.37)	1.59 (0.78, 3.25)	1.41 (0.74, 2.69)
High (>63.37–100.00)	4.59 ** (1.85, 11.38)	2.35 + (0.98, 5.61)
School size		
Small (fewer than 500 students)	0.61 (0.25, 1.48)	0.42 (0.14, 1.27)
Medium (500 to 999 students)	0.89 (0.47, 1.70)	0.27 ** (0.11, 0.65)
Large (1000 or more students)	Referent	Referent
School urbanicity		
Urban	Referent	Referent
Suburban	1.45 (0.74, 2.83)	0.49 + (0.22, 1.10)
Rural	0.42 (0.14, 1.22)	0.91 (0.33, 2.48)
Student-level gender		
Male	Referent	Referent
Female	0.76 (0.50, 1.15)	1.37 (0.79, 2.39)
Student-level race/ethnicity		
White, non-Hispanic	Referent	Referent
Black, non-Hispanic	1.39 (0.50, 3.83)	1.20 (0.51, 2.81)
Hispanic	0.43+ (0.16, 1.17)	1.04 (0.42, 2.57)
Other (including multi-racial)	1.06 (0.42, 2.66)	0.96 (0.36, 2.56)
Household income as a percentage of poverty level		
≤130%	0.98 (0.54, 1.79)	0.34 * (0.14, 0.83)
>130–185%	0.99 (0.47, 2.08)	0.11 ** (0.03, 0.44)
>185%	Referent	Referent
Census region		
West	Referent	Referent
Midwest	0.61 (0.30, 1.26)	0.71 (0.29, 1.73)
South	0.88 (0.42, 1.85)	1.32 (0.55, 3.19)
Northeast	0.49 + (0.21, 1.13)	0.96 (0.34, 2.73)
Student grade (continuous)	0.88 (0.66, 1.17)	0.99 (0.78, 1.26)

Notes: Model was computed taking into account the SNMCS 2014–2015 sampling design, controlling for the variables shown. *n* = 514 middle school students and *n* = 510 high school students. Outcome only counts consumption where item was consumed at school and either (1) consumed at breakfast or lunch or (2) obtained at school. For middle school, water without calories, 100% juice, unflavored skim or 1% milk, and flavored skim milk were counted as compliant (regardless of kcal) as long as they contained no caffeine. For high school, 100% juice, unflavored skim or 1% milk, flavored skim milk, and any of the following with at most 10 kcal were counted as compliant: water, diet carbonated soda, regular carbonated soda, fruit drinks, sports or energy drinks, and tea or coffee. All other beverages were considered non-compliant. SS: Smart Snacks. NH: non-Hispanic. FRPL: free/reduced-price lunch. + *p* < 0.10, * *p* < 0.05, ** *p* < 0.01.

**Table A2:** Logistic regression results predicting student consumption of Smart Snacks non-compliant beverages at the 60 kcal threshold by school level.

Variable	Middle School OR (95% CI)	High School OR (95% CI)
% of à la carte and vending machine beverages sold in school that are SS compliant	1.00 (0.98, 1.01)	0.98 * (0.96, 1.00)
District policy meets SS for à la carte and vending machine beverages	1.02 (0.58, 1.77)	1.31 (0.76, 2.26)
School-level race/ethnicity of students		
≥50% NH White	Referent	Referent
≥50% NH Black	0.15 + (0.02, 1.13)	0.57 (0.12, 2.69)
≥50% Hispanic	0.25 * (0.08, 0.75)	0.45 (0.11, 1.77)
Mixed	0.55 (0.26, 1.15)	0.24 ** (0.09, 0.65)
School-level FRPL eligibility rates		
Low (0.00–37.42)	Referent	Referent
Medium (>37.42–63.37)	1.59 (0.78, 3.25)	1.93 + (0.94, 3.95)
High (>63.37-100.00)	4.59 ** (1.85, 11.38)	3.31 * (1.20, 9.13)
School size		
Small (fewer than 500 students)	0.61 (0.25, 1.48)	0.36 + (0.11, 1.19)
Medium (500 to 999 students)	0.89 (0.47, 1.70)	0.25 ** (0.10, 0.60)
Large (1000 or more students)	Referent	Referent
School urbanicity		
Urban	Referent	Referent
Suburban	1.45 (0.74, 2.83)	0.80 (0.28, 2.34)
Rural	0.42 (0.14, 1.22)	1.44 (0.43, 4.77)
Student-level gender		
Male	Referent	Referent
Female	0.76 (0.50, 1.15)	1.16 (0.66, 2.04)
Student-level race/ethnicity		
White, non-Hispanic	Referent	Referent
Black, non-Hispanic	1.39 (0.50, 3.83)	1.58 (0.66, 3.80)
Hispanic	0.43 + (0.16, 1.17)	1.34 (0.55, 3.27)
Other (including multi-racial)	1.06 (0.42, 2.66)	0.97 (0.35, 2.71)
Household income as a percentage of poverty level		
≤130%	0.98 (0.54, 1.79)	0.35 * (0.13, 0.91)
>130–185%	0.99 (0.47, 2.08)	0.12 ** (0.03, 0.49)
>185%	Referent	Referent
Census region		
West	Referent	Referent
Midwest	0.61 (0.30, 1.26)	0.70 (0.30, 1.65)
South	0.88 (0.42, 1.85)	1.41 (0.65, 3.06)
Northeast	0.49 + (0.21, 1.13)	1.04 (0.41, 2.60)
Student grade (continuous)	0.88 (0.66, 1.17)	0.99 (0.79, 1.23)

Notes: Model was computed taking into account the SNMCS 2014–2015 sampling design, controlling for the variables shown. *n* = 514 middle school students and *n* = 510 high school students. Outcome only counts consumption where item was consumed at school and either (1) consumed at breakfast or lunch or (2) obtained at school. For middle school, water without calories, 100% juice, unflavored skim or 1% milk, and flavored skim milk were counted as compliant as long as they contained no caffeine (regardless of kcal). For high school, 100% juice, unflavored skim or 1% milk, flavored skim milk, and any of the following with at most 60 kcal were counted as compliant: water, diet carbonated soda, regular carbonated soda, fruit drinks, sports or energy drinks, and tea or coffee. All other beverages were considered non-compliant. SS: Smart Snacks. NH: non-Hispanic. FRPL: free-/reduced-price lunch. + *p* < 0.10, * *p* < 0.05, ** *p* < 0.01.

**Table A3:** Survey-weighted characteristics of the analytic sample of students who consumed and did not consume Smart Snacks non-compliant (NC) beverages by school level.

Variable	Middle School		High School (10 kcal Threshold)		High School (60 kcal Threshold)	
	Did Not Consume NC Beverages	Consumed NC Beverages	Did Not Consume NC Beverages	Consumed NC Beverages	Did Not Consume NC Beverages	Consumed NC Beverages
	% or Mean (95% CI)	% or Mean (95% CI)	% or Mean (95% CI)	% or Mean (95% CI)	% or Mean (95% CI)	% or Mean (95% CI)
% of à la carte and vending machine beverages sold in school that are SS compliant (school predictor) (mean)	84.70 (78.25, 91.16)	84.03 (76.49, 91.56)	74.42 (70.52, 78.32)	72.61 (67.00, 78.23)	74.09 (70.17, 78.00)	74.25 (68.97, 79.52)
District policy meets SS for à la carte and vending machine beverages	47.35 (32.81, 62.36)	44.41 (27.99, 62.16)	33.45 (21.82, 47.51)	29.63 (16.28, 47.71)	32.82 (21.36, 46.78)	32.61 (17.85, 51.87)
School-level race/ethnicity of students						
≥50% NH White	58.97 (43.68, 72.70)	63.73 (46.01, 78.37)	69.76 (55.68, 80.90)	75.47 (59.26, 86.68)	69.26 (54.80, 80.72)	79.41 (64.31, 89.19)
≥50% NH Black	3.78 (0.79, 16.25)	4.82 (1.28, 16.46)	4.46 (1.79, 10.65)	6.66 (2.45, 16.88)	4.72 (1.88, 11.35)	5.51 (2.10, 13.65)
≥50% Hispanic	14.18 (6.84, 27.11)	7.88 (3.57, 16.50)	6.70 (2.98, 14.38)	5.43 (2.24, 12.55)	6.50 (2.88, 13.99)	6.39 (2.63, 14.69)
Mixed	23.07 (12.66, 38.28)	23.57 (11.50, 42.26)	19.08 (9.80, 33.86)	12.44 (4.65, 29.26)	19.52 (9.84, 35.01)	8.70 (2.75, 24.27)
School-level FRPL eligibility rates						
Low (0.00–37.42)	38.77 (25.70, 53.70)	39.82 (23.98, 58.12)	58.20 (44.55, 70.69)	59.19 (41.22, 74.99)	58.93 (45.30, 71.30)	55.11 (36.11, 72.73)
Medium (>37.42–63.37)	40.72 (26.84, 56.26)	33.32 (18.89, 51.74)	25.47 (16.22, 37.64)	24.40 (12.50, 42.17)	25.03 (15.92, 37.06)	26.78 (13.46, 46.24)
High (>63.37-100.00)	20.50 (11.89, 33.02)	26.86 (14.81, 43.69)	16.33 (9.23, 27.24)	16.41 (8.50, 29.33)	16.04 (9.05, 26.85)	18.10 (9.35, 32.14)
School size						
Small (fewer than 500 students)	18.66 (9.99, 32.16)	16.72 (7.76, 32.38)	7.74 (3.52, 16.16)	5.92 (1.88, 17.18)	7.50 (3.41, 15.72)	6.97 (2.21, 19.89)
Medium (500 to 999 students)	55.34 (41.06, 68.79)	55.83 (38.16, 72.14)	22.75 (13.32, 36.07)	10.98 (5.01, 22.38)	22.36 (13.10, 35.50)	11.12 (5.10, 22.55)
Large (1000 or more students)	26.00 (16.25, 38.88)	27.45 (14.53, 45.72)	69.52 (56.67, 79.91)	83.10 (70.19, 91.12)	70.13 (57.40, 80.37)	81.91 (68.10, 90.57)
School urbanicity						
Urban	31.75 (19.41, 47.33)	27.82 (14.29, 47.11)	20.52 (11.39, 34.17)	24.62 (13.02, 41.61)	21.41 (11.73, 35.83)	20.18 (10.03, 36.44)
Suburban	43.85 (30.01, 58.72)	57.08 (39.68, 72.90)	56.59 (42.70, 69.51)	44.29 (27.22, 62.83)	56.22 (42.18, 69.32)	44.27 (26.55, 63.57)
Rural	24.40 (13.98, 39.05)	15.10 (7.59, 27.79)	22.89 (13.78, 35.53)	31.09 (15.23, 53.11)	22.37 (13.44, 34.85)	35.55 (17.77, 58.47)
Student-level gender						
Male	48.29 (42.83, 53.78)	55.42 (47.20, 63.36)	52.35 (46.64, 58.00)	42.00 (30.48, 54.47)	51.61 (45.97, 57.21)	44.49 (31.77, 57.97)
Female	51.71 (46.22, 57.17)	44.58 (36.64, 52.80)	47.65 (42.00, 53.36)	58.00 (45.53, 69.52)	48.39 (42.79, 54.03)	55.51 (42.03, 68.23)
Student-level race/ethnicity						
White, non-Hispanic	52.76 (41.96, 63.30)	59.07 (47.26, 69.92)	56.16 (48.15, 63.86)	56.56 (44.54, 67.86)	56.60 (48.97, 63.92)	54.07 (41.67, 65.99)
Black, non-Hispanic	10.45 (5.50, 18.95)	15.48 (8.21, 27.28)	13.01 (8.62, 19.15)	16.33 (9.25, 27.19)	12.81 (8.48, 18.88)	18.08 (10.55, 29.23)
Hispanic	25.94 (17.51, 36.62)	12.24 (7.05, 20.41)	20.08 (14.50, 27.12)	18.79 (10.75, 30.77)	19.72 (14.36, 26.46)	20.66 (11.56, 34.15)
Other (including multi-racial)	10.86 (7.47, 15.51)	13.20 (7.16, 23.08)	10.76 (7.37, 15.44)	8.32 (3.78, 17.33)	10.88 (7.59, 15.36)	7.19 (2.93, 16.60)
Household income as a percentage of poverty level						
≤130%	30.73 (23.46, 39.10)	26.19 (17.76, 36.84)	24.20 (18.40, 31.14)	15.61 (8.74, 26.33)	23.87 (18.15, 30.71)	16.03 (8.56, 28.01)
>130–185%	8.99 (6.12, 13.02)	9.08 (5.15, 15.52)	10.96 (7.64, 15.48)	2.03 (0.54, 7.38)	10.63 (7.36, 15.11)	2.39 (0.63, 8.66)
>185%	60.28 (51.24, 68.67)	64.72 (53.80, 74.30)	64.84 (56.34, 72.49)	82.36 (71.33, 89.76)	65.50 (57.05, 73.08)	81.58 (69.29, 89.68)
Census region						
West	15.03 (7.67, 27.35)	12.42 (6.45, 22.59)	12.35 (5.83, 24.29)	9.94 (3.62, 24.50)	12.23 (5.80, 23.96)	10.24 (3.87, 24.44)
Midwest	27.94 (16.48, 43.24)	28.08 (15.02, 46.32)	32.33 (20.24, 47.35)	26.15 (13.49, 44.56)	32.21 (20.16, 47.20)	25.77 (12.46, 45.83)
South	44.34 (29.94, 59.76)	46.93 (29.99, 64.61)	41.84 (28.54, 56.44)	49.83 (31.69, 68.01)	42.05 (28.62, 56.76)	50.02 (30.96, 69.06)
Northeast	12.69 (5.96, 25.00)	12.57 (5.27, 27.09)	13.48 (6.31, 26.49)	14.08 (4.86, 34.49)	13.52 (6.33, 26.55)	13.97 (5.18, 32.57)

Notes: Weighted percentages or means were computed taking into account the SNMCS 2014–2015 sampling design. *n* = 514 middle school students and *n* = 510 high school students. Consumption measure only counts consumption where item was consumed at school and either (1) consumed at breakfast or lunch or (2) obtained at school. NC: non-compliant. NH: non-Hispanic. FRPL: free/reduced-price lunch.
